# Comparative effectiveness of non‐pharmacological therapies of traditional Chinese medicine on overall cognitive function in elderly with mild cognitive impairment: a systematic evaluation and network meta‐analysis

**DOI:** 10.1002/alz70858_099011

**Published:** 2025-12-25

**Authors:** Tingting YIN

**Affiliations:** ^1^ Nanjing University of chinese medicine, Nanjing, Jiangsu, China

## Abstract

**Background:**

Cognitive, exercise and Traditional Chinese Medicine (TCM) non‐pharmacological therapies can be effective in patients with mild cognitive impairment(MCI). However, because of lack of resources, TCM non‐pharmacological therapies are being used more frequently worldwide. We aimed to evaluate the comparative effectiveness of TCM non‐pharmacological therapies in treating overall cognitive function in elderly people with MCI.

**Methods:**

In this systematic review and network meta‐analysis, we searched the Cochrane Central Register of Controlled Trials, PubMed, Web of Science, Embase, CNKI, Vip, Wanfang, and China Biomedical Literature from database inception to October 31, 2023, to identify published and unpublished randomised controlled trials. We included studies comparing TCM non‐pharmacologic treatment or placebo as monotherapy for mild cognitive impairment. The primary outcome was the efficacy of cognitive function (i.e., overall cognitive function as measured by any neuropsychological scale). We used paired and network meta‐analyses with random effects to estimate standardized mean differences (SMDs) and 95% confidence intervals.

**Results:**

We included 34 trials in the systematic review and in the network meta‐analysis, comprising 9 interventions (specifically hand acupuncture, electroacupuncture, warm acupuncture, moxibustion, medicated wire moxibustion, transcutaneous electrical stimulation of acupoints, acupuncture point burrowing, auricular acupoint therapy, acupressure, and the control group was divided into three categories, namely, the Western medication group, the sham acupuncture group, and the minimum‐treatment group, which consisted of health education, blank control group) and 2779 subjects. The results of the best probability ranking showed that electroacupuncture was the most effective in terms of the MMSE scale, followed by warm acupuncture and moxibustion, and moxibustion was the second most effective after electroacupuncture and warm acupuncture and moxibustion (electroacupuncture SMD 3.66, 95% CI[2.52,4.80], warm acupuncture and moxibustion SMD 3.78, 95% CI [2.08,5.48], moxibustion SMD 3.47, 95% CI [2.44,4.50 ]). From the MoCA scale, acupressure was the most effective, followed by transcutaneous electrical acupoint stimulation (acupressure SMD 5.03, 95%CI[3.36,6.70], transcutaneous electrical acupoint stimulation SMD 3.68, 95%CI [2.26,5.10]).

**Conclusion:**

Current evidence suggests that electroacupuncture and acupressure are more advantageous in external TCM non‐pharmacological therapies for MCI, but observation of long‐term efficacy is lacking. These results should cautiously serve the evidence‐based clinical practice.

